# The Role of Autophagy in Liver Epithelial Cells and Its Impact on Systemic Homeostasis

**DOI:** 10.3390/nu11040827

**Published:** 2019-04-11

**Authors:** Luana Tomaipitinca, Sara Mandatori, Romina Mancinelli, Federico Giulitti, Simonetta Petrungaro, Viviana Moresi, Antonio Facchiano, Elio Ziparo, Eugenio Gaudio, Claudia Giampietri

**Affiliations:** 1 Department of Anatomical, Histological, Forensic Medicine and Orthopedic Sciences, Sapienza University of Rome, 00161 Rome, Italy; luana.tomaipitinca@uniroma1.it (L.T.); sara.mandatori@uniroma1.it (S.M.); romina.mancinelli@uniroma1.it (R.M.); federico.giulitti@uniroma1.it (F.G.); simonetta.petrungaro@uniroma1.it (S.P.); viviana.moresi@uniroma1.it (V.M.); elio.ziparo@uniroma1.it (E.Z.); eugenio.gaudio@uniroma1.it (E.G.); 2 Laboratory of Molecular Oncology, Istituto Dermopatico dell’Immacolata, IDI-IRCCS, 00167 Rome, Italy; a.facchiano@idi.it

**Keywords:** autophagy, liver, lipid droplets, metabolism, oxidative stress, cancer

## Abstract

Autophagy plays a role in several physiological and pathological processes as it controls the turnover rate of cellular components and influences cellular homeostasis. The liver plays a central role in controlling organisms’ metabolism, regulating glucose storage, plasma proteins and bile synthesis and the removal of toxic substances. Liver functions are particularly sensitive to autophagy modulation. In this review we summarize studies investigating how autophagy influences the hepatic metabolism, focusing on fat accumulation and lipids turnover. We also describe how autophagy affects bile production and the scavenger function within the complex homeostasis of the liver. We underline the role of hepatic autophagy in counteracting the metabolic syndrome and the associated cardiovascular risk. Finally, we highlight recent reports demonstrating how the autophagy occurring within the liver may affect skeletal muscle homeostasis as well as different extrahepatic solid tumors, such as melanoma.

## 1. Autophagy Machinery

Autophagy is an evolutionarily conserved process. Autophagic bodies formation was observed for the first time by electronic microscopy in the middle of the twentieth century [1]. Ever since autophagy deep involvement in physiological and pathological contexts has been emerging, thus prompting researchers to unravel the molecular platform operating within this mechanism [2].

Autophagy is a quality-control process aimed at eliminating old or damaged cellular components. It contributes to the turnover of macromolecules such as proteins [3], lipids (lipophagy) [4,5] and nucleic acids (RNautophagy, DNautophagy) [6]. In addition, selective forms of autophagy of whole organelles have been characterized, such as the autophagy of mitochondria (mitophagy) [7], ribosomes (ribophagy) [8] and endoplasmic reticulum (ER-phagy) [9]. Moreover, autophagy is involved in the defense against pathogens invasion through the selective degradation of foreign material, namely, xenophagy [10]. Therefore, working autophagic machinery is pivotal within metabolic processes to properly recycle cellular elements and maintain homeostasis [11].

**Figure 1:**
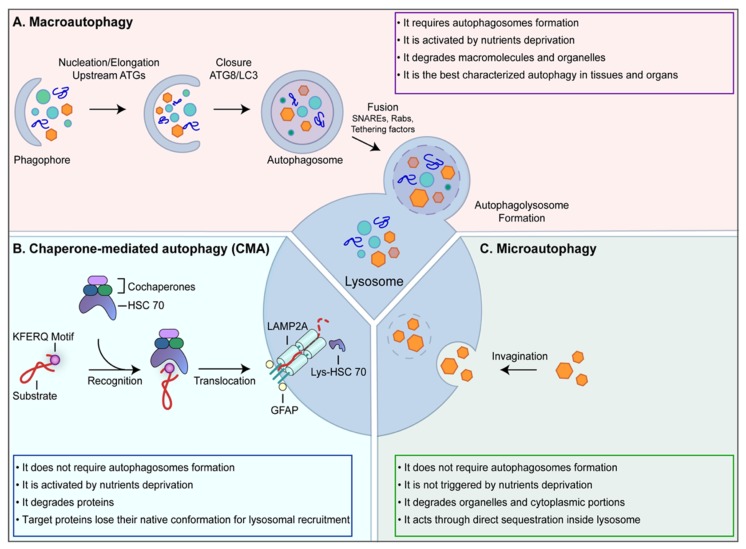
Autophagy machinery. Autophagy is a quality-control process aimed at eliminating old or damaged cellular components. (**A**) Macroautophagy is the most common form of autophagy. It requires different sequential steps leading to the formation of autophagosomes. Following the fusion between autophagosomes and lysosome, the cargo is degraded and the resulting macromolecules are released back into the cytosol to be recycled; (**B**) chaperone-mediated autophagy (CMA) contributes to the cellular homeostasis by recycling amino acids upon proteins degradation and by eliminating abnormal or damaged proteins. CMA targets are recognized by this molecular machinery as they contain the KFERQ motif; (**C**) Microautophagy is involved in organelles and cytoplasmic portions turnover through direct sequestration inside lysosomes.

Three types of autophagy have been identified: macroautophagy, chaperone-mediated autophagy (CMA) and microautophagy (Figure 1).

The main difference between the three processes consists of the cargo-recruitment modality. While in microautophagy and CMA autophagic, substrates are incorporated directly in lysosomes, in macroautophagy, the formation of a double-membrane structure, namely phagophore or isolation membrane, mediates substrates incorporation. In higher eukaryotes, autophagic vesicles originate from membranous sources, such as the endoplasmic reticulum (ER) or mitochondria, and their nucleation point is referred to as the omegasome. Phagofores develop into autophagosomes, which eventually fuse with lysosomes [12]. However, it is also evident that the three autophagic mechanisms work in an interdependent way. They can be individually activated at the same time and they can, at least partially, compensate each other [13,14].

Macroautophagy is the best characterized among the three types, so the term “autophagy” is often used to indicate “macroautophagy” (Figure 1A). Macroautophagy is generally triggered by stress conditions, such as nutrient deprivation, and it mediates degradation of cytoplasmic components to provide energy to the cells, thus, it may be considered as a recycling process able to generate energy from waste material [12]. The molecular switch responsible for nutrient-dependent autophagy modulation is the mammalian Target Of Rapamycin kinase (mTOR), whose enzymatic activity promotes cell growth and protein synthesis [15]. mTOR is the main negative regulator of autophagy as it directly inactivates Unc-51-Like Kinase 1 (ULK1), thus inhibiting autophagy [16]. Once autophagy is triggered, the phosphatidylinositol-3-phosphate kinase (PI3K) class III complex is recruited at the omegasome through an ULK1-dependent process to mediate the synthesis of phosphatidylinositol-3-phosphate (PtdIns3P) [17]. The core unit of this complex is made by vacuolar protein sorting 34 (Vps34) kinase, Vps15 and the coiled-coil, Moesin-like BCL2-interacting protein (Beclin-1) [18]. PtdIns3P is the signal recognized by the WD-repeat domain phosphoinositide-Interacting Proteins (WIPI) proteins [19], which bind both the PtdIns3P and the molecular factors belonging to the microtubule-associated protein light chain 3 (MAPLC3, shortly LC3) proteins family maturation machinery, thus mediating LC3 protein placement at the omegasome [20]. LC3 cleavage and lipidation [21] are pivotal for phagophore expansion, autophagic cargo recruitment by binding autophagic cargo-carriers, such as p62, and vesicle closure around the cargo to form autophagosomes [22]. These vesicles are transported along microtubules by the dynein-dynactin motor complex to fuse with lysosomes [23], resulting in the formation of a single-membrane large vesicle, called autophagolysosome, where hydrolytic enzymes catalyze cargo degradation [23].

The second type of autophagy, named CMA, is activated to degrade damaged proteins and, like macroautophagy, to provide energy when low levels of nutrients are available (Figure 1B). The selective recruitment of the target proteins by the CMA machinery occurs through the exposure of the pentapeptide motif: KFERQ, which is recognized by the molecular chaperon Heat shock cognate protein of 70 kDa (Hsc70) [24]. CMA targets are then transferred across the lysosomal membranes, without the formation of autophagosomes. While a stable channel at the lysosomal membrane has not been identified yet, it is known that the lysosome-associated membrane protein type 2A (LAMP-2A) [25] is involved in the process, as a positively charged tail of LAMP-2A binds CMA substrates. To be translocated inside the lysosome, proteins lose their native structural conformation, but the molecular mechanism responsible for this unfolding process has not been unraveled yet. Finally, the CMA substrates are degraded by lysosomal proteases [26].

Microautophagy, similarly to CMA, does not require autophagosomes formation (Figure 1C). It is active under basal conditions and it may co-occur with macroautophagy. As a difference with the other two types of autophagy, microautophagy is not triggered by nutrient deprivation. It is involved in organelles and cytoplasmic portions turnover through direct sequestration inside lysosomes, where specific enzymes degrade the substrates. The underlying mechanisms are only partially known; it is emerging that microautophagy may occur through different engulfment modalities of the substrates: lysosomal protrusion, lysosomal invagination and endosomal invagination, even if there is no clear separation between the molecular machinery characterizing the different pathways [27,28].

## 2. Cell Types within the Liver

**Figure 2:**
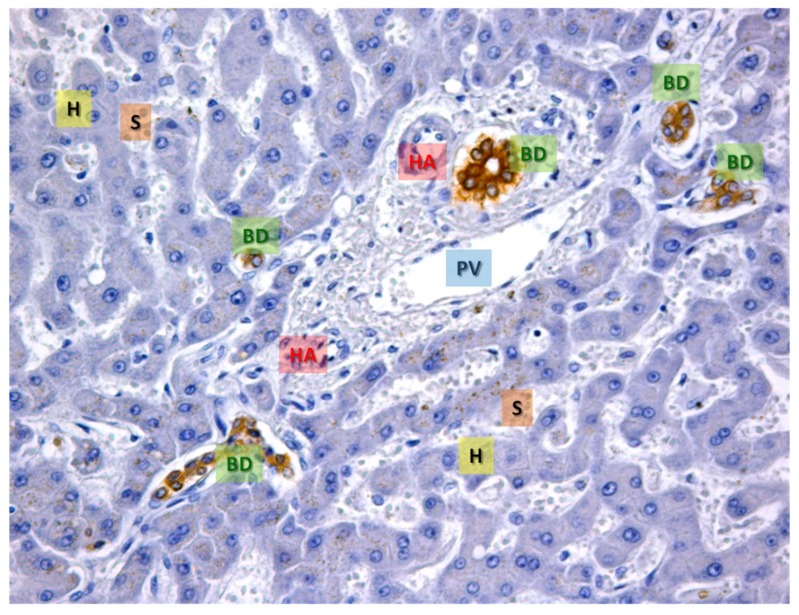
The representative area of normal hepatic tissue. Immunohistochemistry for CK-7, a specific cytokeratin of the biliary epithelium, that is stained in brown (BD). In blue the hepatocytes are evident (H), the white spaces are the sinusoids (S). In the center, a portal space is evident with the typical branches of the portal vein (PV), hepatic artery (HA) and several bile ducts (BD) cut on different planes (transverse or sagittal). OM 20x.

The liver is essential in keeping the appropriate balance between body anabolism and catabolism as it performs a broad range of tissue-specific functions including the intermediary metabolism, detoxification, plasma proteins synthesis and bile acid formation [29]. The liver lobule is formed by epithelial and non-epithelial cells (Figure 2).

**Hepatocytes.** Hepatocytes and cholangiocytes are the most abundant epithelial cells in the liver, representing almost 85% of the total organ. They originate and differentiate from the embryonic liver stem cells, namely, hepatoblasts [30]. Hepatocytes are multifaced and polynucleated cells; they fulfill many physiological functions, such as the metabolism of glucose, lipids and amino acids, bile secretion, bilirubin and drugs detoxification and serum protein synthesis. As epithelial cells, hepatocytes are polarized cells with a basal or vascular domain adjacent to sinusoidal endothelial cells in order to allow efficient substance exchanges with the bloodstream. Moreover, each hepatocyte has a lateral or biliary domain in contact with another hepatocyte to form the origin of the biliary system: the biliary canaliculi (BC). The vascular pole allows efficient substance exchange with the bloodstream as it contains small pinocytosis vesicles and numerous microvilli, which increase the membrane surface in contact with the plasma. Hepatocyte cytoplasm contains specific components. Lysosomes, peroxisomes and Golgi vesicles usually localize close to the biliary pole, as they perform secretory functions. Mitochondria are numerous with well-developed ridges and they undergo modifications in number and in shape following the cellular functional needs. Large cytoplasm portions are dedicated to glycogen (a glucose polymer) and fats deposit, and the percentage depends on individual diets and on the digestion phase [31,32].

**Cholangiocytes.** Cholangiocytes are the cells lining the biliary ducts, therefore, they are also known as biliary cells. They are mitotically inactive cells, but numerous factors, such as damage, destruction of normal cell-matrix interactions and release of inflammatory cytokines can re-activate their proliferation. Cholangiocytes have copious microvilli and one primary cilium, working as a sensor between the bile flow and the cells. These cells form the intrahepatic and extrahepatic bile ducts (IHBD and EHBD) to link the liver with the duodenum and to drain bile juice. Cholangiocytes dimensions depend on their localization. “Small” cholangiocytes form biliary ductules walls while “large” cholangiocytes form structure ducts. These cells also exert a key role in modulating bile composition [33,34].

As mentioned above, hepatocytes and cholangiocytes form about 85% of the liver volume while the remaining 15% is occupied by non-parenchymal cells, which are localized in the sinusoidal compartment. In fact, the walls of hepatic sinusoids are formed by four different cell types: Kupffer cells (KC), hepatic stellate cells (HSC, also known as fat-storing cells or Ito cells), pit cells and sinusoidal endothelial cells (SEC). It has been demonstrated that under pathophysiological conditions, many hepatocyte activities are regulated by substances released from neighboring non-epithelial cells [35].

**Kupffer cells.** Kupffer cells belong to the monocytic/macrophage system as they possess phagocytic power to digest bacteria and viruses and to present antigens. Their development starts in the bone marrow and it is completed once they are located in the lumen of the liver sinusoids. Kupffer cells are the first cells exposed to material absorbed from the gastrointestinal tract [36].

**HSC**. HSC is located at the level of the perisinusoidal space, namely, the space of Disse, in close contact with hepatocytes and endothelial cells lining the hepatic sinusoid. In a healthy liver, HSC cells are quiescent cells, presenting cytoplasmic lipid droplets containing vitamin A in the form of retinyl palmitate. Upon hepatic damage, these cells start proliferating. Consequently, the amount of vitamin A progressively decreases while the synthesis and deposition of the extracellular matrix begins, leading to fibrosis and cirrhosis [37].

**Pit cells.** Pit cells are natural killer lymphocytes, containing specific granules. They depend on and localize close to Kupffer cells as they both exert defensive functions. More in detail, pit cells perform anti-tumor surveillance through the exocytosis of perforin/granzyme-containing granuli, induce apoptosis in target cells via death receptors and release cytokines to increase other immune cells activity [38].

**SEC.** SEC are epithelial cells with a flattened shape, an oval nucleus and poor cytoplasm. They may be considered a functional unit between the hepatocytes and the blood. Indeed, they take part in several liver functions and pathologies. These cells present fenestration complexes, which allow contact between the blood and hepatocytes microvilli in the space of Disse [39].

**HPC.** Another cell type worth mentioning is hepatic stem/progenitors cells (HPC), also known as oval cells, which localize at the canals of Hering, at the beginning of the biliary system [40]. These cells are responsible for the regenerative capability of the liver tissue after partial hepatectomy or chemical injury. In detail, these cells express several stem markers, such as CD44 and CD133, but also alpha-fetoprotein (AFP), albumin, cytokeratin (CK-7) and CK-19, indicating a partial commitment to hepatocytes and cholangiocytes [41].

The following part of this review focuses on the role autophagic processes play in regulating and preserving liver homeostasis, summarizing the latest reports about autophagy relevance in hepatic epithelial cell types. Within the liver, the process of macroautophagy appears to be the most important to maintain hepatic homeostasis to inhibit spontaneous tumorigenesis [42].

## 3. Autophagy Involvement in Lipid Droplets Turnover

As previously mentioned, hepatocytes are key controllers of the glucose and lipid metabolism. Hepatocytes can store glucose as glycogen, which serves as a reserve pool, ready for quick mobilization to meet sudden needs. Furthermore, hepatocytes can store neutral lipids, i.e., cellular triglycerides (TAGs) and cholesterol esters, as energy reserve source. Hepatic TAGs homeostasis is maintained through a fine balance between lipid import/synthesis and lipid secretion/lipolysis. Dysregulation of these processes leads to excess TAGs content in the liver and, consequently, to fatty liver disease [43].

**Figure 3:**
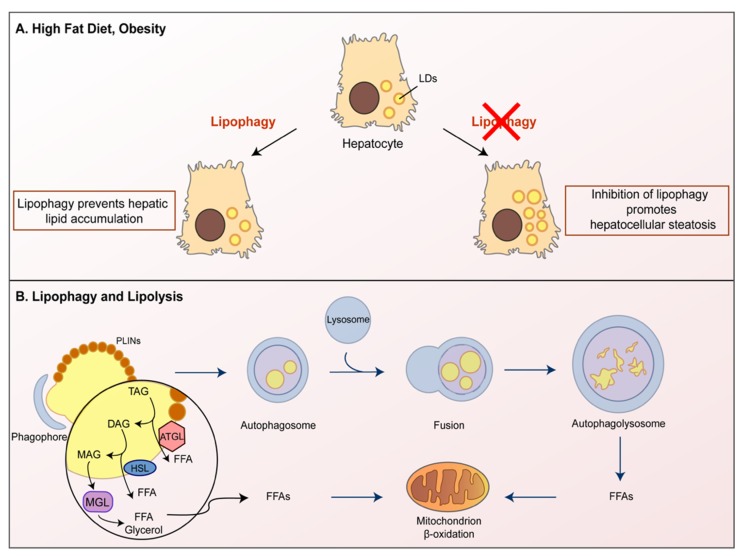
The autophagy involvement in lipid droplets turnover. Autophagy plays a key role in lipid droplets (LDs) metabolism in hepatocytes. (**A**) Autophagy breaks LDs inside lysosomes in a process termed lipophagy. Lipophagy can prevent lipid accumulation in hepatocytes, while the inhibition of lipophagy promotes LDs accumulation, resulting in hepatocellular steatosis. (**B**) Lipolysis is a process mediated by three lipases: ATGL, HSL and MGL. ATGL controls the crosstalk between lipolysis and autophagy as it regulates TAG turnover. ATGL promotes lipophagy to facilitate LD catabolism leading to the generation of Free Fatty Acids (FFAs), which are broken down by the β-oxidation process.

Neutral lipids are mainly contained within lipid droplets (LDs), specialized organelles provided with a phospholipid monolayer, likely derived from the ER [44]. In the presence of nutritional request and to support increased cell growth, LDs can be readily catabolized, thus supporting energetic purposes. Autophagy was shown to play a key role in LDs metabolism in hepatocytes (Figure 3). In detail, autophagy target LDs breakdown in lysosomes in a process termed lipophagy. In the presence of high-fat diet and obesity, LDs accumulation occurs leading to hepatic steatosis. Autophagy/lipophagy counteracts such lipid accumulation [45], while the inhibition of autophagy/lipophagy promotes hepatocellular steatosis (Figure 3A).

While many details underlying the regulation of autophagic LDs turnover in the liver remain unknown, the small GTPase Ras-related protein in the brain (Rab-7) has been recently identified as a central regulator of hepatocellular lipophagy. Indeed, Rab-7 primes LDs for autophagic degradation, thus, promoting interaction between autophagosomal LDs and lysosomes [46]. LDs are surrounded by perilipins (PLINs), proteins encoded by 5 different genes, PLIN1 to PLIN5 [47,48]; PLIN2 is ubiquitously expressed, and its expression levels correlate with TAGs accumulation and LDs density. Interestingly, Plin2−/− mice show a strong reduction in TAGs content, related to hepatic TAGs degradation through autophagy [49].

An additional crosstalk protein mediating lipolysis and autophagy is the adipose triglyceride lipase (ATGL), a crucial hepatic lipase regulating TAGs turnover (Figure 3B). ATGL is an enzyme known to hydrolyze TAGs, thus, producing Free Fatty Acids (FFAs). Mobilization of stored FFAs is mediated by the activity of three different TAGs hydrolases: ATGL, the hormone-sensitive lipase (HSL) and monoglyceride lipase (MGL). The complex action of ATGL, HSL and MGL controls the complete TAGs hydrolysis [50]. ATGL starts the process of triglyceride (TAG) metabolism by hydrolyzing TAG into diacylglycerol (DAG) and FFA. Subsequently, HSL breaks down DAG into monoacylglycerol (MAG) and FFA. Finally, MAG is further broken down into FFA and glycerol by MGL [51]. Remarkably, ATGL can induce autophagy via sirtuin1 (SIRT1), a NAD-dependent deacetylase, able to deacetylate autophagy-related gene proteins. SIRT1 can both deacetylate ATG5, ATG7, and ATG8 and activate the transcription factors Forkhead box O (FoxO) FoxO1 and FoxO3 [52,53], ultimately promoting autophagy induction. It is noteworthy that hepatic SIRT1 deletion significantly increases reactive oxygen species (ROS) levels in the liver, leading to severe hepatic oxidative stress and eventually fatty liver diseases [54]. ATGL activity also promotes peroxisome proliferator-activated receptor-α (PPAR-α) and PPAR-γ coactivator 1-α (PGC-1α) signaling. PPAR-α is a highly expressed transcription factor in the liver and it regulates enzymes crucial for fatty acid oxidation since it increases the cellular capability to mobilize and catabolize fatty acids [55]. PPAR-α-mediated autophagic pathway induction has been demonstrated [56] and PPAR-α activation is able to counteract the normal autophagy suppression occurring in the fed state [57]. Other studies also showed that SIRT1 mediates the effects of ATGL on PPAR-α/PGC-1α signaling, thus, regulating both autophagy and oxidative metabolism in the liver [58]. Finally, autophagy is required for ATGL to promote LDs degradation and the subsequent oxidation of fatty acids, as ATGL and LC3 interaction mediates the recruitment of ATGL to LDs, building a synergistic link between lipolysis and lipophagy [59].

## 4. The Autophagy at the Crossroad between Oxidative Stress, Lipids Accumulation and Cell Death in a Hypercaloric Diet

**Figure 4:**
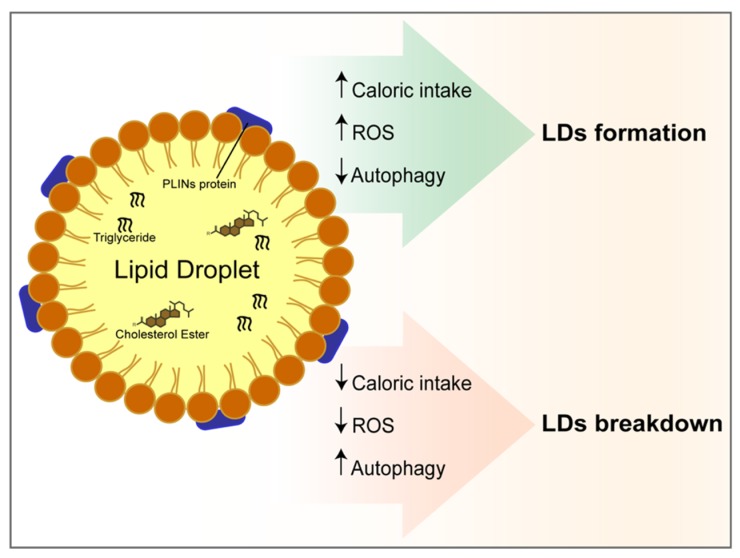
Autophagy at the crossroad between oxidative stress, lipids accumulation and cell death in a hypercaloric diet. Lipid droplets consist of a hydrophobic core of neutral lipids, surrounded by a phospholipid monolayer characterized by perilipins (PLINs) proteins. The core of LDs is composed of triglycerides and cholesterol esters. The figure shows the positive and negative correlation between reactive oxygen species (ROS), autophagy and diet with LDs formation or breakdown.

A hypercaloric diet with high fat or carbohydrate intake increases the lipid content within hepatic cells (Figure 4). Under such a condition, polyunsaturated fatty acid induces stronger hepatic fat accumulation than saturated fatty acids [60]. Chronic FFAs and glucose overload stimulate ROS production. Remarkably increased ROS production from an oxidative metabolism, including superoxide anion (O_2_−) and hydroxyl radical (OH−) production, as well as the reduction of antioxidant enzymes, resulting in neutral lipid production and aggregation. These events lead to LDs formation within hepatocytes depending on the up-regulation of PLIN2 [61,62]. Furthermore, ROS-induced PLIN2 upregulation might also contribute to the LD accumulation by counteracting autophagy promotion, therefore reducing LDs breakdown [63]. Moreover, hepatic expression of ATG7 is decreased in obese ob/ob mice liver [64,65]. Consequently, the autophagy induction in the liver represents a buffer system to moderate LDs accumulation and ROS production, thus mitigating oxidative stress.

## 5. Liver, Diet Habits and Autophagy

Alcohol abuse influences autophagic flux. An alcohol-containing diet alters hepatic autophagy, reducing the lysosome number within hepatocytes [66,67]. In chronic ethanol abuse, liver injury is associated with steatosis and progress toward fibrosis and cirrhosis, resulting in alcoholic liver disease. Although such chronic ethanol administration is associated with increased macroautophagy, this is selective for damaged mitochondria and for part of LDs, leading to a long-lived protein accumulation within hepatocytes [68]. Furthermore, ethanol-induced liver damage is exacerbated by dietary fructose [69], likely by increasing steatosis and oxidative stress. As an example, related to diet habit, methionine is an essential amino acid whose optimal level strictly depends on diet components. Methionine reduction leads to the relevant metabolic changes during protein restriction. In detail, methionine dietary restriction leads to a decrease in mitochondrial ROS production and oxidative stress, thus, affecting autophagy [70]. Different pharmacological treatments or lifestyles may stimulate autophagy (e.g., rapamycin, polyphenols, exercise, fasting), preventing LDs increase and fatty liver [71,72]. Herbal medicine may represent a source of interesting dietary supplements to treat lipid metabolic disorders. For instance, glycycoumarin, a major coumarin compound isolated from licorice, can re-activate impaired autophagy, thus protecting against non-alcoholic fatty liver disease (NAFLD) [73]. On the other hand, it must also be considered that mobilization and degradation of intracellular LDs via lipophagy can lead to ferroptosis in hepatocytes [74]. Ferroptosis is a form of non-apoptotic cell death, where excess free iron leads to oxidative damage and ROS production, inducing lipid peroxidation and subsequent cell death [74,75]. This highlights a possible antioxidant role for LDs in cell death, underlying the complex contribution of autophagy to hepatocytes metabolism and LDs accumulation.

A number of diet-supplied molecules have been shown to directly affect autophagy with beneficial actions in the liver. An incomplete list includes,

- the purple sweet potato color, showing proautophagy action [76] and beneficial effects on metabolic syndrome [77];
- caffeic acid, found in many vegetables, an autophagy inducer which ameliorates hepatic steatosis [78];
- resveratrol, a non-flavonoid polyphenol found in grapes and red wine, showing proautophagy action and reducing lipid metabolism disorder [79];
- curcumin, a polyphenolic compound present in Curcuma longa, an antioxidant, apoptosis-inhibitor and autophagy-inducer with a protective effect on hepatocellular carcinoma [80] and under evaluation in clinical trials on NAFLD [81].
  The role diet plays in autophagy control is currently investigated and a large debate is ongoing on the actual role that diets and healthy foods may have on the health [82].

## 6. The Role of Autophagy on Biliary Epithelium Differentiation and Homeostasis

**Figure 5:**
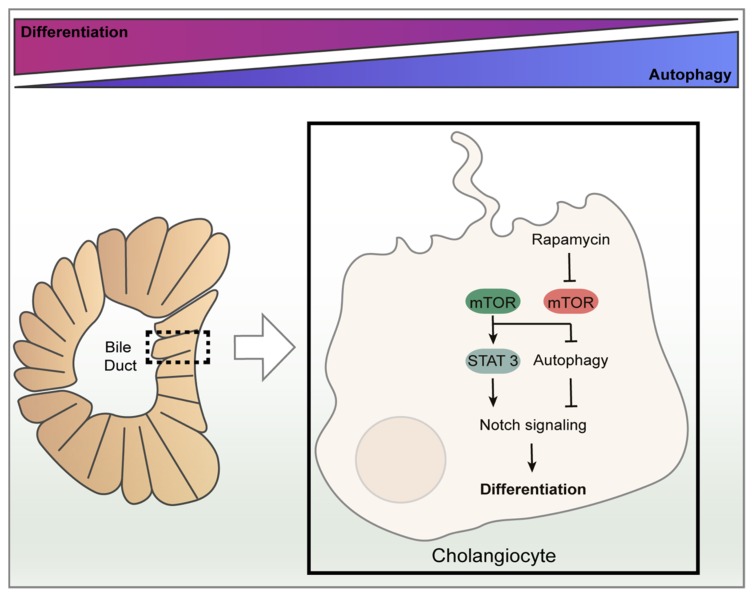
The role of autophagy on biliary epithelium differentiation and homeostasis. Autophagy is involved in the maintenance and functions of progenitory cells in the liver (HPC). It negatively correlates with biliary tree formation, it decreases in the early stages of HPC development while it increases in the late stages. HPC differentiation to cholangiocyte is controlled by the Notch signaling pathway. In cholangiocytes, the under nutrient-rich condition or absence of rapamycin, mTOR blocks autophagy resulting in an increased Notch-STAT3 cascade signaling pathway and promoting cellular differentiation. On the contrary, autophagy induction via rapamycin (an mTOR inhibitor) or nutrient deprivation attenuates the Notch signaling pathway resulting in a reduced biliary differentiation.

As previously described, cholangiocytes are the epithelial lining cells within the biliary system, which are differentiated from HPC cells through mechanisms still not completely understood [83]. Adult stem cells usually present high autophagic activity under physiological conditions, as constitutive autophagy plays an important role in the maintenance of cellular homeostasis; on the contrary, in mature cells, autophagy occurrence is usually linked to stressful conditions [84]. It is recently emerging that autophagy controls the maintenance and functions of progenitory cells in the liver as well. Therefore, blocking autophagy in those cell types, particularly in HPC, decreases their differentiation competence and function, promoting the upregulation of cell cycle factors p53 and p21 and sensitizing cells to etoposide-induced senescence [85]. Furthermore, the knockdown of Atg5 and Beclin-1 decreases the autophagic activity within HPC and negatively controls their stemness [86]. Moreover, it was demonstrated that autophagy negatively correlates with biliary differentiation. In detail, HPC differentiation is controlled by positive feedback between the transcription factor STAT3 and Notch signaling pathway, thus targetting the *Sox9* promoter, a known regulator of biliary development [87,88]. Autophagy decreases in the early stages of the hepatic progenitors’ differentiation, maintaining a low level in the late stages. Indeed, in cholangiocytes, autophagy induction via rapamycin (an mTOR inhibitor) or nutrient deprivation attenuates biliary differentiation as it results in the downregulation of factors belonging to Notch signaling (Figure 5) [89].

Autophagy in cholangiocytes is also triggered by ER stress, a cellular condition caused by the accumulation of unfolded proteins. ER stress results in the unfolded protein response (UPR), i.e., the activation of three receptors named PRKR-like endoplasmic reticulum kinase (PERK), activating transcription factor 6 (ATF6) and inositol-requiring enzyme 1 (hIRE1p). Downstream these three receptor activations signal cascades initiate at aiming to cope with the stress source. It is well established that some UPR mediators can directly participate in autophagy by connecting these two cellular mechanisms [90,91,92].

An in vitro study showed that some bile acids such as glycochenodeoxycholic acid (GCDC), together with starvation and oxidative stress, can induce deregulated autophagy and the abnormal expression of mitochondrial proteins in cholangiocytes. As a consequence, cellular senescence is induced in the biliary tree, impairing its functions in bile secretion and reabsorption. On the contrary, pretreatment with other types of bile acids, such as ursodeoxycholic acid (UDCA) and Tauro-UDCA, a chemical chaperone that increases the ER adaptive capacity, significantly decreases ER stress, autophagy and cellular senescence induced by GCDC [91]. Accordingly, UDCA is currently used as a standard treatment in primary biliary cirrhosis (PBC) since it acts as an anti-cholestatic, anti-fibrotic and anti-proliferative agent [93]. Another piece of evidence of the relevance of autophagy in the biliary tree is represented by the G protein-coupled bile acid receptor 1 (GPBAR1 or TGR5), a cAMP-linked bile acid receptor involved in polycystic liver disease (PLD). TGR5 may be considered one of the main inducers of cAMP-regulated autophagy, contributing to hepatic cystogenesis [94]. Remarkably both molecular and pharmacological inhibition of autophagy prevents such a hepatic disease [93]. More studies are needed to clarify the emerging leading role of autophagy on the biliary epithelium in maintaining the balance between cell death and proliferation in these essential cells in the liver, especially considering that 10–40% of mice with Atg5 deletion develop hepatic benign tumors [83].

## 7. The Role of Autophagy in Endothelial Cells Maintainance

Sinusoidal endothelial liver cells (SEC) play important physiological roles in mediating liver filtration and scavenger functions. The maintenance of the SEC phenotype, associated with their fenestrae, is critical to maintaining liver homeostasis [43]. Pivotal studies revealed that autophagy is implicated in maintaining the endothelial cell’s metabolism and preventing cardiovascular diseases. Autophagy dysfunction in endothelial cells can contribute to the pathogenesis of atherosclerosis by influencing nitric oxide (NO) production [53]. Indeed, it was demonstrated that intact autophagic flux keeps regular endothelial nitric oxide synthase (eNOS) activity. eNOS regulates NO production that, in turn, affects autophagy induction as high levels of NO correlate with the inhibition of autophagosomes biogenesis [95,96,97].

**Figure 6:**
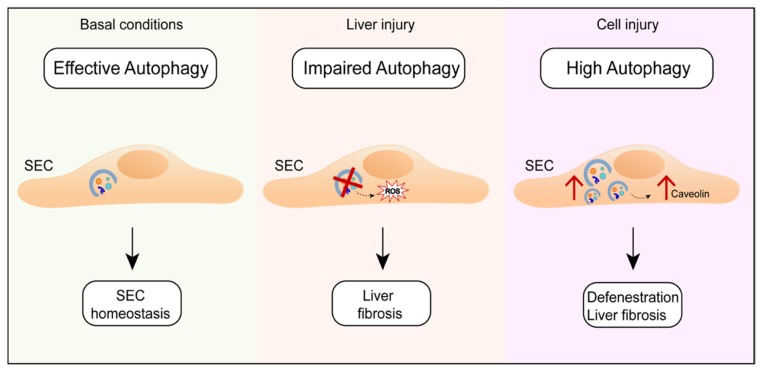
The role of autophagy in endothelial cells maintenance. Autophagy plays an important role in the regulation of the Sinusoidal endothelial liver cells (SEC) phenotype. In fact, under basal conditions, autophagy maintains SEC homeostasis. During liver injury, autophagy impairment increases oxidative stress and leads to liver fibrosis. On the other hand, a high autophagy rate induces caveolin-1 degradation, thus leading to SEC defenestration and liver fibrosis.

As far as liver cells are concerned, although autophagy has been implicated in the regulation of hepatocytes and cholangiocytes, its role in the regulation of SEC functions remains largely unknown. It was recently demonstrated by a pharmacological and a genetic approach that autophagy plays an important role in controlling the SEC phenotype. In fact, autophagy impairment within SECs during the liver injury results in increased oxidative stress, which exacerbates liver fibrosis [98]. At the same time, excessive autophagy activation may lead to caveolin-1 degradation, thus worsening the SEC defenestration and ultimately promoting fibrosis [99]. Therefore, dysregulated or uncoordinated autophagy is linked to endothelial cell injury and liver fibrosis (Figure 6) [100].

## 8. The Role of Liver Autophagy in Preserving Systemic Homeostasis Counteracting Cancer Growth

Liver functions are tightly related to the control of whole-body energetics. A change of autophagy in the liver leads to altered glucose- and amino acid-blood levels and may impact skeletal muscle catabolic pathways upon starvation [101], contributing to metabolic disorders associated to obesity and fatty liver. Visceral obesity increases the amount of free fatty acids in the body. In the liver, in turn, higher fatty acids levels increase the production of glucose, triglycerides and very low-density lipoproteins. Obesity, dyslipidemia and insulin resistance are often associated with a metabolic syndrome and a marked increase in cardiovascular risk. When insulin resistance occurs, target organs, including the liver, do not properly respond to insulin and the glucose uptake from the bloodstream is less effective. As an adaptive response, higher blood glucose levels lead to higher insulin levels. Insulin has a lipolytic effect, thus, high insulin levels prime further increases in fatty acid levels. It has been observed that autophagy regulates the function of pancreatic β cells and other insulin targets, including the liver, thus counteracting dyslipidemia [102].

Dyslipidemia has been strongly linked to colon, renal, gallbladder, pancreatic, endometrial and postmenopausal breast cancers, as well as to oesophageal adenocarcinoma and leukemia [103,104]. Obesity has been associated with increased mortality in several cancer types; however, interestingly enough, in metastatic melanoma, obesity has been associated with improved progression-free and overall survival, and this correlation is mainly observed in male patients treated with targeted- or immune-therapy [105]. Conversely, in uveal melanoma, insulin resistance, metabolic syndrome and dyslipidemia seem to promote the growth of uveal melanocytic tumors, contributing to its aggressive clinical course [106]. As recently pointed out, obesity is considered a major preventable cancer risk factor for many cancer types. For instance, obesity has shown a positive correlation with melanoma risk in men and different correlations in pre- vs post-menopausal women; furthermore, adipocytes and obesity have been reported to induce growth and aggressive behavior of melanoma, indicating that adipose tissue as a key player in melanoma progression [107]. On the contrary, a weakly increased body mass index may represent a protective factor inducing improved outcome in patients under cancer treatment, a phenomenon known as “obesity paradox” [108].

According to what was reported above, obesity and fat metabolism play a key role in the way cells and the whole organism counteract cancer growth and progression; fat metabolism is finely controlled in each cell-type, but the liver is the most important organ in regulating the whole body cholesterol metabolism, mainly via autophagy. Indeed, autophagy, by maintaining the proper cellular organelle function, is directly involved in cholesterol accumulation [109]. The cholesterol metabolism appears to play a key role in cancer setup, nevertheless with controversial evidence. Recently the expression of genes controlling cholesterol synthesis has been inversely related to survival in sarcoma, acute myeloid leukemia and melanoma [110] and some evidence indicates that melanoma cells are cells particularly sensitive to the inhibition of intracellular cholesterol transport [111].

A recent report indicates a novel metabolic pathway linking cholesterol to histamine metabolism, showing the dendrogenin A metabolite with strong antitumor and autophagy-inducing actions [112].

Dyslipidemia also causes fat infiltration and aberrant muscle-derived cell differentiation negatively affecting skeletal muscle homeostasis [113]. Obesity, insulin resistance and dyslipidemia are considered risk factors for developing non-alcoholic fatty liver disease, a chronic condition characterized by liver fat accumulation and inflammation. Importantly, NAFLD is associated with a cardiometabolic syndrome and the majority of NAFLD patients die because of cardiovascular diseases [114]. Moreover, progressive increases in the intrahepatic triglyceride content correlate with the progressive impairment of insulin action in the liver, skeletal muscle and adipose tissue in nondiabetic obese subjects [115], confirming that the alteration in liver fat storage can systemically affect numerous organs, including muscles.

Notably, liver enzymes participating in both glycolysis and lipolysis can be degraded by autophagy, further influencing the energy balance [116]. Moreover, genetic studies on autophagic proteins clarify the role of autophagy in promoting lipophagy, the selective autophagic LDs degradation [64]. Defects in lipophagy promote hepatic diseases and participate in the onset and progression of systemic disorders, including atherosclerosis, obesity, metabolic syndrome and organ dysfunction in aging [117]. All these studies demonstrate that a fine liver autophagy regulation underlines metabolism and whole-body homeostasis.

On the other way around, dietary (high-fat diet) or genetic (ob/ob mouse) models of obesity show reduced liver autophagy that alters insulin signaling in these models [64,65]. Importantly, the restoration of insulin signaling can be achieved by correcting the autophagic defect [65]. Similar to obesity, a decrease in liver macroautophagy has been found with age [118], reducing liver ability to mobilize intracellular lipids and contributing to the systemic alteration of the lipid metabolism, a hallmark of the metabolic syndrome. Controlling liver macroautophagy may represent an attractive antiaging therapy that is able to prevent or delay the onset of the metabolic syndrome. Coherently, several pharmacological approaches that efficiently increase the life span in vertebrates, such as rapamycin, resveratrol, or spermidine [119], trigger macroautophagy.

Recent data also demonstrate that high ferritin expression can enhance cell growth and improve resistance to oxidative stress in metastatic melanoma cells [120]. Ferritin is stored in many cell types, including hepatocytes and, in the presence of liver damage from any cause, the ferritin level increases in the blood [121]. Remarkably, it has been demonstrated that autophagy leads to ferritin degradation and this also results in the free iron release, leading to oxidative damage and ROS-dependent cell death [122]. This particular kind of autophagy called “ferritinophagy” may both control the ferritin level and induce ferroptosis through the degradation of ferritin and the release of iron [123]. Both these events (i.e., the reduction of ferritin level and the increase of ferroptosis) play an important role in inhibiting different cancer types, such as melanoma, hepatocellular carcinoma, pancreatic carcinoma, prostate cancer and breast cancer [124], further highlighting the crucial role of autophagy in systemic homeostasis. Moreover, elevated serum ferritin levels have been registered in Amyotrophic Lateral Sclerosis patients [125] and are correlated with reduced survival [126].

The control autophagy exerted on the cytochrome p450-dependent drug metabolism is an additional way through which the liver metabolism and liver-localized autophagy may affect the systemic response to cancer treatment as well as a drug’s toxicity. Different cytochrome p-450 inhibitors are known to block autophagic flux in hepatocytes and to affect the drug’s toxicity in hepatocytes [127], and autophagy has been found to be related to p450-dependent chemoresistance, at least in some cases [128]. As recently reviewed, autophagy may directly affect the drug metabolism and drug-toxicity, thus controlling the response to therapy and disease progression [129].

## 9. Conclusions

In conclusion, autophagic processes occurring within the liver are key actors regulating the organ metabolism under physiological conditions by mediating the TAG metabolism in hepatocytes, regulating liver development and controlling HPC stemness, cholangiocyte differentiation and SEC functions. Impaired liver autophagy thus contributes to changes in the hepatic oxidative stress and induction of liver steatosis and fibrosis in a cell-autonomous manner.

While it is difficult to discriminate the precise role that the liver plays in systemic diseases, as several organs may contribute to the disease progression, several studies demonstrate the impact of liver autophagy on body metabolism and its consequences on other districts. Indeed, liver autophagy directly impacts glucose- and amino acids-blood levels, thereby regulating the whole-body metabolism and systemically affecting numerous tissues, including adipose tissue, skeletal and cardiac muscles. Obesity is considered one of the major cancer risk factors for many cancer types, generally promoting cancer progression and correlating with increased mortality, linking an alteration in liver autophagy to tumorigenesis. Moreover, liver autophagy is altered in aging.

Importantly, by correcting the autophagic defect in the liver, insulin signaling can be rescued. Therefore, the manipulation of liver autophagy represents an attractive therapy that is able to prevent or delay the metabolic syndrome and counteract aging, or even cancer progression. Coherently, numerous pharmacological treatments and lifestyle approaches aimed to ameliorate obesity or to exert anti-tumor activity may stimulate autophagy or may be affected by autophagy. Understanding the fine impact of liver autophagy on other organs by determining soluble factors and modulated signals, is needed to develop more effective pharmacological approaches.

## References

[1] Deter R.L., Baudhuin P., De Duve C. (1967). Participation of lysosomes in cellular autophagy induced in rat liver by glucagon. J. Cell Biol..

[2] Tsukada M., Ohsumi Y. (1993). Isolation and characterization of autophagy-defective mutants of *Saccharomyces cerevisiae*. FEBS Lett..

[3] Zientara-Rytter K., Subramani S. (2019). The Roles of Ubiquitin-Binding Protein Shuttles in the Degradative Fate of Ubiquitinated Proteins in the Ubiquitin-Proteasome System and Autophagy. Cells.

[4] Giampietri C., Petrungaro S., Cordella M., Tabolacci C., Tomaipitinca L., Facchiano A., Eramo A., Filippini A., Facchiano F., Ziparo E. (2017). Lipid Storage and Autophagy in Melanoma Cancer Cells. Int. J. Mol. Sci..

[5] Zhang Z., Yao Z., Chen Y., Qian L., Jiang S., Zhou J., Shao J., Chen A., Zhang F., Zheng S. (2018). Lipophagy and liver disease: New perspectives to better understanding and therapy. Biomed. Pharmacother..

[6] Hase K., Fujiwara Y., Kikuchi H., Aizawa S., Hakuno F., Takahashi S., Wada K., Kabuta T. (2015). RNautophagy/DNautophagy possesses selectivity for RNA/DNA substrates. Nucleic Acids Res..

[7] Sedlackova L., Korolchuk V.I. (2018). Mitochondrial quality control as a key determinant of cell survival. Biochim. Biophys. Acta Mol. Cell Res..

[8] Beau I., Esclatine A., Codogno P. (2008). Lost to translation: When autophagy targets mature ribosomes. Trends Cell Biol..

[9] Grumati P., Dikic I., Stolz A. (2018). ER-phagy at a glance. J. Cell Sci..

[10] Kwon D.H., Song H.K. (2018). A Structural View of Xenophagy, a Battle between Host and Microbes. Mol. Cells.

[11] Mizushima N. (2018). A brief history of autophagy from cell biology to physiology and disease. Nat. Cell Biol..

[12] Mizushima N. (2007). Autophagy: Process and function. Genes Dev..

[13] Kon M., Cuervo A.M. (2010). Chaperone-mediated autophagy in health and disease. FEBS Lett..

[14] Klionsky D.J., Abdelmohsen K., Abe A., Abedin M.J., Abeliovich H., Acevedo Arozena A., Adachi H., Adams C.M., Adams P.D., Adeli K. (2016). Guidelines for the use and interpretation of assays for monitoring autophagy (3rd edition). Autophagy.

[15] Roux P.P., Topisirovic I. (2018). Signaling Pathways Involved in the Regulation of mRNA Translation. Mol. Cell. Biol..

[16] Hosokawa N., Hara T., Kaizuka T., Kishi C., Takamura A., Miura Y., Iemura S., Natsume T., Takehana K., Yamada N. (2009). Nutrient-dependent mTORC1 association with the ULK1-Atg13-FIP200 complex required for autophagy. Mol. Biol. Cell.

[17] Paquette M., El-Houjeiri L., Pause A. (2018). mTOR Pathways in Cancer and Autophagy. Cancers.

[18] Boutouja F., Brinkmeier R., Mastalski T., El Magraoui F., Platta H.W. (2017). Regulation of the Tumor-Suppressor BECLIN 1 by Distinct Ubiquitination Cascades. Int. J. Mol. Sci..

[19] D’Arcangelo D., Giampietri C., Muscio M., Scatozza F., Facchiano F., Facchiano A. (2018). WIPI1, BAG1, and PEX3 Autophagy-Related Genes Are Relevant Melanoma Markers. Oxid. Med. Cell. Longev..

[20] Dooley H.C., Razi M., Polson H.E., Girardin S.E., Wilson M.I., Tooze S.A. (2014). WIPI2 links LC3 conjugation with PI3P, autophagosome formation, and pathogen clearance by recruiting Atg12-5-16L1. Mol. Cell.

[21] Ohsumi Y. (2001). Molecular dissection of autophagy: Two ubiquitin-like systems. Nat. Rev. Mol. Cell Biol..

[22] Lee Y.K., Lee J.A. (2016). Role of the mammalian ATG8/LC3 family in autophagy: Differential and compensatory roles in the spatiotemporal regulation of autophagy. BMB Rep..

[23] Nakamura S., Yoshimori T. (2017). New insights into autophagosome-lysosome fusion. J. Cell Sci..

[24] Chiang H.L., Terlecky S.R., Plant C.P., Dice J.F. (1989). A role for a 70-kilodalton heat shock protein in lysosomal degradation of intracellular proteins. Science.

[25] Rout A.K., Strub M.P., Piszczek G., Tjandra N. (2014). Structure of transmembrane domain of lysosome-associated membrane protein type 2a (LAMP-2A) reveals key features for substrate specificity in chaperone-mediated autophagy. J. Biol. Chem..

[26] Kaushik S., Cuervo A.M. (2018). The coming of age of chaperone-mediated autophagy. Nat. Rev. Mol. Cell Biol..

[27] Todde V., Veenhuis M., van der Klei I.J. (2009). Autophagy: Principles and significance in health and disease. Biochim. Biophys. Acta.

[28] Oku M., Sakai Y. (2018). Three Distinct Types of Microautophagy Based on Membrane Dynamics and Molecular Machineries. Bioessays.

[29] Takiguchi M. (1998). The C/EBP family of transcription factors in the liver and other organs. Int. J. Exp. Pathol..

[30] Kmiec Z. (2001). Cooperation of liver cells in health and disease. Adv. Anat. Embryol. Cell Biol..

[31] Fraczek J., Bolleyn J., Vanhaecke T., Rogiers V., Vinken M. (2013). Primary hepatocyte cultures for pharmaco-toxicological studies: At the busy crossroad of various anti-dedifferentiation strategies. Arch. Toxicol..

[32] Gaudio E., Onori P., Franchitto A., Sferra R., Riggio O. (1997). Liver metabolic zonation and hepatic microcirculation in carbon tetrachloride-induced experimental cirrhosis. Dig. Dis. Sci..

[33] Tanimizu N., Mitaka T. (2016). Morphogenesis of liver epithelial cells. Hepatol. Res..

[34] Sato K., Meng F., Giang T., Glaser S., Alpini G. (2018). Mechanisms of cholangiocyte responses to injury. Biochim. Biophys. Acta Mol. Basis Dis..

[35] Bouwens L., De Bleser P., Vanderkerken K., Geerts B., Wisse E. (1992). Liver cell heterogeneity: Functions of non-parenchymal cells. Enzyme.

[36] Dixon L.J., Barnes M., Tang H., Pritchard M.T., Nagy L.E. (2013). Kupffer cells in the liver. Compr. Physiol..

[37] Ren L., Qi K., Zhang L., Bai Z., Ren C., Xu X., Zhang Z., Li X. (2019). Glutathione Might Attenuate Cadmium-Induced Liver Oxidative Stress and Hepatic Stellate Cell Activation. Biol. Trace Elem. Res..

[38] Nakatani K., Kaneda K., Seki S., Nakajima Y. (2004). Pit cells as liver-associated natural killer cells: Morphology and function. Med. Electron Microsc..

[39] Sorensen K.K., Simon-Santamaria J., McCuskey R.S., Smedsrod B. (2015). Liver Sinusoidal Endothelial Cells. Compr. Physiol..

[40] Gera S., Ettel M., Acosta-Gonzalez G., Xu R. (2017). Clinical features, histology, and histogenesis of combined hepatocellular-cholangiocarcinoma. World J. Hepatol..

[41] Castorina S., Luca T., Torrisi A., Privitera G., Panebianco M. (2008). Isolation of epithelial cells with hepatobiliary phenotype. Ital. J. Anat. Embryol..

[42] Weiskirchen R., Tacke F. (2019). Relevance of Autophagy in Parenchymal and Non-Parenchymal Liver Cells for Health and Disease. Cells.

[43] Allaire M., Rautou P.E., Codogno P., Lotersztajn S. (2019). Autophagy in liver diseases: Time for translation?. J. Hepatol..

[44] Schulze R.J., Rasineni K., Weller S.G., Schott M.B., Schroeder B., Casey C.A., McNiven M.A. (2017). Ethanol exposure inhibits hepatocyte lipophagy by inactivating the small guanosine triphosphatase Rab7. Hepatol. Commun..

[45] Sharma L., Lone N.A., Knott R.M., Hassan A., Abdullah T. (2018). Trigonelline prevents high cholesterol and high fat diet induced hepatic lipid accumulation and lipo-toxicity in C57BL/6J mice, via restoration of hepatic autophagy. Food Chem. Toxicol..

[46] Schroeder B., Schulze R.J., Weller S.G., Sletten A.C., Casey C.A., McNiven M.A. (2015). The small GTPase Rab7 as a central regulator of hepatocellular lipophagy. Hepatology.

[47] Brasaemle D.L. (2007). Thematic review series: Adipocyte biology. The perilipin family of structural lipid droplet proteins: Stabilization of lipid droplets and control of lipolysis. J. Lipid Res..

[48] Brasaemle D.L., Wolins N.E. (2012). Packaging of fat: An evolving model of lipid droplet assembly and expansion. J. Biol. Chem..

[49] Tsai T.H., Chen E., Li L., Saha P., Lee H.J., Huang L.S., Shelness G.S., Chan L., Chang B.H. (2017). The constitutive lipid droplet protein PLIN2 regulates autophagy in liver. Autophagy.

[50] Zechner R., Kienesberger P.C., Haemmerle G., Zimmermann R., Lass A. (2009). Adipose triglyceride lipase and the lipolytic catabolism of cellular fat stores. J. Lipid Res..

[51] Liu X., Liang Y., Song R., Yang G., Han J., Lan Y., Pan S., Zhu M., Liu Y., Wang Y. (2018). Long non-coding RNA NEAT1-modulated abnormal lipolysis via ATGL drives hepatocellular carcinoma proliferation. Mol. Cancer.

[52] Lee I.H., Cao L., Mostoslavsky R., Lombard D.B., Liu J., Bruns N.E., Tsokos M., Alt F.W., Finkel T. (2008). A role for the NAD-dependent deacetylase Sirt1 in the regulation of autophagy. Proc. Natl. Acad. Sci. USA.

[53] Jiang F. (2016). Autophagy in vascular endothelial cells. Clin. Exp. Pharmacol. Physiol..

[54] Pfluger P.T., Herranz D., Velasco-Miguel S., Serrano M., Tschop M.H. (2008). Sirt1 protects against high-fat diet-induced metabolic damage. Proc. Natl. Acad. Sci. USA.

[55] Di Leo L., Vegliante R., Ciccarone F., Salvatori I., Scimeca M., Bonanno E., Sagnotta A., Grazi G.L., Aquilano K., Ciriolo M.R. (2018). Forcing ATGL expression in hepatocarcinoma cells imposes glycolytic rewiring through PPAR-alpha/p300-mediated acetylation of p53. Oncogene.

[56] Jiao M., Ren F., Zhou L., Zhang X., Zhang L., Wen T., Wei L., Wang X., Shi H., Bai L. (2014). Peroxisome proliferator-activated receptor alpha activation attenuates the inflammatory response to protect the liver from acute failure by promoting the autophagy pathway. Cell Death Dis..

[57] Lee J.M., Wagner M., Xiao R., Kim K.H., Feng D., Lazar M.A., Moore D.D. (2014). Nutrient-sensing nuclear receptors coordinate autophagy. Nature.

[58] Khan S.A., Sathyanarayan A., Mashek M.T., Ong K.T., Wollaston-Hayden E.E., Mashek D.G. (2015). ATGL-catalyzed lipolysis regulates SIRT1 to control PGC-1alpha/PPAR-alpha signaling. Diabetes.

[59] Vegliante R., Di Leo L., Ciccarone F., Ciriolo M.R. (2018). Hints on ATGL implications in cancer: Beyond bioenergetic clues. Cell Death Dis..

[60] Sharp K.P.H., Schultz M., Coppell K.J. (2018). Is non-alcoholic fatty liver disease a reflection of what we eat or simply how much we eat?. JGH Open.

[61] Jin Y., Tan Y., Chen L., Liu Y., Ren Z. (2018). Reactive Oxygen Species Induces Lipid Droplet Accumulation in HepG2 Cells by Increasing Perilipin 2 Expression. Int. J. Mol. Sci..

[62] Sekiya M., Hiraishi A., Touyama M., Sakamoto K. (2008). Oxidative stress induced lipid accumulation via SREBP1c activation in HepG2 cells. Biochem. Biophys. Res. Commun..

[63] Filomeni G., De Zio D., Cecconi F. (2015). Oxidative stress and autophagy: The clash between damage and metabolic needs. Cell Death Differ..

[64] Singh R., Kaushik S., Wang Y., Xiang Y., Novak I., Komatsu M., Tanaka K., Cuervo A.M., Czaja M.J. (2009). Autophagy regulates lipid metabolism. Nature.

[65] Yang L., Li P., Fu S., Calay E.S., Hotamisligil G.S. (2010). Defective hepatic autophagy in obesity promotes ER stress and causes insulin resistance. Cell Metab..

[66] Thomes P.G., Trambly C.S., Fox H.S., Tuma D.J., Donohue T.M. (2015). Acute and Chronic Ethanol Administration Differentially Modulate Hepatic Autophagy and Transcription Factor EB. Alcohol. Clin. Exp. Res..

[67] Kharbanda K.K., McVicker D.L., Zetterman R.K., Donohue T.M. (1995). Ethanol consumption reduces the proteolytic capacity and protease activities of hepatic lysosomes. Biochim. Biophys. Acta.

[68] Ding W.X., Li M., Chen X., Ni H.M., Lin C.W., Gao W., Lu B., Stolz D.B., Clemens D.L., Yin X.M. (2010). Autophagy reduces acute ethanol-induced hepatotoxicity and steatosis in mice. Gastroenterology.

[69] Thomes P.G., Benbow J.H., Brandon-Warner E., Thompson K.J., Jacobs C., Donohue T.M., Schrum L.W. (2017). Dietary fructose augments ethanol-induced liver pathology. J. Nutr. Biochem..

[70] Sanchez-Roman I., Barja G. (2013). Regulation of longevity and oxidative stress by nutritional interventions: Role of methionine restriction. Exp. Gerontol..

[71] Mancinelli R., Carpino G., Petrungaro S., Mammola C.L., Tomaipitinca L., Filippini A., Facchiano A., Ziparo E., Giampietri C. (2017). Multifaceted Roles of GSK-3 in Cancer and Autophagy-Related Diseases. Oxid. Med. Cell. Longev..

[72] Cingolani F., Czaja M.J. (2016). Regulation and Functions of Autophagic Lipolysis. Trends Endocrinol. Metab..

[73] Zhang E., Yin S., Song X., Fan L., Hu H. (2016). Glycycoumarin inhibits hepatocyte lipoapoptosis through activation of autophagy and inhibition of ER stress/GSK-3-mediated mitochondrial pathway. Sci. Rep..

[74] Bogdan A.R., Miyazawa M., Hashimoto K., Tsuji Y. (2016). Regulators of Iron Homeostasis: New Players in Metabolism, Cell Death, and Disease. Trends Biochem. Sci..

[75] Bai Y., Meng L., Han L., Jia Y., Zhao Y., Gao H., Kang R., Wang X., Tang D., Dai E. (2018). Lipid storage and lipophagy regulates ferroptosis. Biochem. Biophys. Res. Commun..

[76] Zhuang J., Lu J., Wang X., Wang X., Hu W., Hong F., Zhao X.X., Zheng Y.L. (2019). Purple sweet potato color protects against high-fat diet-induced cognitive deficits through AMPK-mediated autophagy in mouse hippocampus. J. Nutr. Biochem..

[77] Wang X., Zhang Z.F., Zheng G.H., Wang A.M., Sun C.H., Qin S.P., Zhuang J., Lu J., Ma D.F., Zheng Y.L. (2017). The Inhibitory Effects of Purple Sweet Potato Color on Hepatic Inflammation Is Associated with Restoration of NAD(+) Levels and Attenuation of NLRP3 Inflammasome Activation in High-Fat-Diet-Treated Mice. Molecules.

[78] Kim H.M., Kim Y., Lee E.S., Huh J.H., Chung C.H. (2018). Caffeic acid ameliorates hepatic steatosis and reduces ER stress in high fat diet-induced obese mice by regulating autophagy. Nutrition.

[79] Ding S., Jiang J., Zhang G., Bu Y., Zhang G., Zhao X. (2017). Resveratrol and caloric restriction prevent hepatic steatosis by regulating SIRT1-autophagy pathway and alleviating endoplasmic reticulum stress in high-fat diet-fed rats. PLoS ONE.

[80] Elmansi A.M., El-Karef A.A., Shishtawy M., Eissa L.A. (2017). Hepatoprotective Effect of Curcumin on Hepatocellular Carcinoma Through Autophagic and Apoptic Pathways. Ann. Hepatol..

[81] Yan S., Huda N., Khambu B., Yin X.M. (2017). Relevance of autophagy to fatty liver diseases and potential therapeutic applications. Amino Acids.

[82] Willett W., Rockstrom J., Loken B., Springmann M., Lang T., Vermeulen S., Garnett T., Tilman D., DeClerck F., Wood A. (2019). Food in the Anthropocene: The EAT-Lancet Commission on healthy diets from sustainable food systems. Lancet.

[83] Han Y., Onori P., Meng F., DeMorrow S., Venter J., Francis H., Franchitto A., Ray D., Kennedy L., Greene J. (2014). Prolonged exposure of cholestatic rats to complete dark inhibits biliary hyperplasia and liver fibrosis. Am. J. Physiol. Gastrointest. Liver Physiol..

[84] Salemi S., Yousefi S., Constantinescu M.A., Fey M.F., Simon H.U. (2012). Autophagy is required for self-renewal and differentiation of adult human stem cells. Cell Res..

[85] Cheng Y., Wang B., Zhou H., Dang S., Jin M., Shi Y., Hao L., Yang Z., Zhang Y. (2015). Autophagy is Required for the Maintenance of Liver Progenitor Cell Functionality. Cell. Physiol. Biochem..

[86] Chen N., Karantza V. (2011). Autophagy as a therapeutic target in cancer. Cancer Biol. Ther..

[87] Zong Y., Panikkar A., Xu J., Antoniou A., Raynaud P., Lemaigre F., Stanger B.Z. (2009). Notch signaling controls liver development by regulating biliary differentiation. Development.

[88] Hildebrand D., Uhle F., Sahin D., Krauser U., Weigand M.A., Heeg K. (2018). The Interplay of Notch Signaling and STAT3 in TLR-Activated Human Primary Monocytes. Front. Cell. Infect. Microbiol..

[89] Zeng J., Jing Y., Shi R., Pan X., Lai F., Liu W., Li R., Gao L., Hou X., Wu M. (2016). Autophagy regulates biliary differentiation of hepatic progenitor cells through Notch1 signaling pathway. Cell Cycle.

[90] Gonzalez-Rodriguez A., Mayoral R., Agra N., Valdecantos M.P., Pardo V., Miquilena-Colina M.E., Vargas-Castrillon J., Lo Iacono O., Corazzari M., Fimia G.M. (2014). Impaired autophagic flux is associated with increased endoplasmic reticulum stress during the development of NAFLD. Cell Death Dis..

[91] Conti S., Petrungaro S., Marini E.S., Masciarelli S., Tomaipitinca L., Filippini A., Giampietri C., Ziparo E. (2016). A novel role of c-FLIP protein in regulation of ER stress response. Cell Signal..

[92] Marini E.S., Giampietri C., Petrungaro S., Conti S., Filippini A., Scorrano L., Ziparo E. (2015). The endogenous caspase-8 inhibitor c-FLIPL regulates ER morphology and crosstalk with mitochondria. Cell Death Differ..

[93] Sasaki M., Nakanuma Y. (2017). Bile Acids and Deregulated Cholangiocyte Autophagy in Primary Biliary Cholangitis. Dig. Dis..

[94] Masyuk A.I., Masyuk T.V., Lorenzo Pisarello M.J., Ding J.F., Loarca L., Huang B.Q., LaRusso N.F. (2018). Cholangiocyte autophagy contributes to hepatic cystogenesis in polycystic liver disease and represents a potential therapeutic target. Hepatology.

[95] Montagna C., Rizza S., Maiani E., Piredda L., Filomeni G., Cecconi F. (2016). To eat, or NOt to eat: S-nitrosylation signaling in autophagy. FEBS J..

[96] Guo F., Li X., Peng J., Tang Y., Yang Q., Liu L., Wang Z., Jiang Z., Xiao M., Ni C. (2014). Autophagy regulates vascular endothelial cell eNOS and ET-1 expression induced by laminar shear stress in an ex vivo perfused system. Ann. Biomed. Eng..

[97] Yang Q., Li X., Li R., Peng J., Wang Z., Jiang Z., Tang X., Peng Z., Wang Y., Wei D. (2016). Low Shear Stress Inhibited Endothelial Cell Autophagy Through TET2 Downregulation. Ann. Biomed. Eng..

[98] Ruart M., Chavarria L., Camprecios G., Suarez-Herrera N., Montironi C., Guixe-Muntet S., Bosch J., Friedman S.L., Garcia-Pagan J.C., Hernandez-Gea V. (2018). Impaired endothelial autophagy promotes liver fibrosis by aggravating the oxidative stress response during acute liver injury. J. Hepatol..

[99] Luo X., Wang D., Zhu X., Wang G., You Y., Ning Z., Li Y., Jin S., Huang Y., Hu Y. (2018). Autophagic degradation of caveolin-1 promotes liver sinusoidal endothelial cells defenestration. Cell Death Dis..

[100] Boteon Y.L., Laing R., Mergental H., Reynolds G.M., Mirza D.F., Afford S.C., Bhogal R.H. (2017). Mechanisms of autophagy activation in endothelial cell and their targeting during normothermic machine liver perfusion. World J. Gastroenterol..

[101] Ezaki J., Matsumoto N., Takeda-Ezaki M., Komatsu M., Takahashi K., Hiraoka Y., Taka H., Fujimura T., Takehana K., Yoshida M. (2011). Liver autophagy contributes to the maintenance of blood glucose and amino acid levels. Autophagy.

[102] Stefanadi E.C., Dimitrakakis G., Antoniou C.K., Challoumas D., Punjabi N., Dimitrakaki I.A., Punjabi S., Stefanadis C.I. (2018). Metabolic syndrome and the skin: A more than superficial association. Reviewing the association between skin diseases and metabolic syndrome and a clinical decision algorithm for high risk patients. Diabetol. Metab. Syndr..

[103] Key T.J., Spencer E.A. (2007). Carbohydrates and cancer: An overview of the epidemiological evidence. Eur. J. Clin. Nutr..

[104] Dobbins M., Decorby K., Choi B.C. (2013). The Association between Obesity and Cancer Risk: A Meta-Analysis of Observational Studies from 1985 to 2011. ISRN Prev. Med..

[105] McQuade J.L., Daniel C.R., Hess K.R., Mak C., Wang D.Y., Rai R.R., Park J.J., Haydu L.E., Spencer C., Wongchenko M. (2018). Association of body-mass index and outcomes in patients with metastatic melanoma treated with targeted therapy, immunotherapy, or chemotherapy: A retrospective, multicohort analysis. Lancet Oncol..

[106] Sevim D.G., Kiratli H. (2016). Serum adiponectin, insulin resistance, and uveal melanoma: Clinicopathological correlations. Melanoma Res..

[107] Clement E., Lazar I., Muller C., Nieto L. (2017). Obesity and melanoma: Could fat be fueling malignancy?. Pigment Cell Melanoma Res..

[108] Hayes A.J., Larkin J. (2018). BMI and outcomes in melanoma: More evidence for the obesity paradox. Lancet Oncol..

[109] Wang Y., Ding W.X., Li T. (2018). Cholesterol and bile acid-mediated regulation of autophagy in fatty liver diseases and atherosclerosis. Biochim. Biophys. Acta Mol. Cell Biol. Lipids.

[110] Kuzu O.F., Noory M.A., Robertson G.P. (2016). The Role of Cholesterol in Cancer. Cancer Res..

[111] Kuzu O.F., Gowda R., Sharma A., Robertson G.P. (2014). Leelamine mediates cancer cell death through inhibition of intracellular cholesterol transport. Mol. Cancer Ther..

[112] Poirot M., Silvente-Poirot S. (2018). The tumor-suppressor cholesterol metabolite, dendrogenin A, is a new class of LXR modulator activating lethal autophagy in cancers. Biochem. Pharmacol..

[113] Masouminia M., Gelfand R., Kovanecz I., Vernet D., Tsao J., Salas R., Castro K., Loni L., Rajfer J., Gonzalez-Cadavid N.F. (2018). Dyslipidemia Is a Major Factor in Stem Cell Damage Induced by Uncontrolled Long-Term Type 2 Diabetes and Obesity in the Rat, as Suggested by the Effects on Stem Cell Culture. J. Sex. Med..

[114] Lim S., Taskinen M.R., Boren J. (2018). Crosstalk between nonalcoholic fatty liver disease and cardiometabolic syndrome. Obes. Rev..

[115] Korenblat K.M., Fabbrini E., Mohammed B.S., Klein S. (2008). Liver, muscle, and adipose tissue insulin action is directly related to intrahepatic triglyceride content in obese subjects. Gastroenterology.

[116] Schneider J.L., Suh Y., Cuervo A.M. (2014). Deficient chaperone-mediated autophagy in liver leads to metabolic dysregulation. Cell Metab..

[117] Singh R., Cuervo A.M. (2012). Lipophagy: Connecting autophagy and lipid metabolism. Int. J. Cell Biol..

[118] Cuervo A.M. (2008). Autophagy and aging: Keeping that old broom working. Trends Genet..

[119] Kaushik S., Singh R., Cuervo A.M. (2010). Autophagic pathways and metabolic stress. Diabetes Obes. Metab..

[120] Baldi A., Lombardi D., Russo P., Palescandolo E., De Luca A., Santini D., Baldi F., Rossiello L., Dell’Anna M.L., Mastrofrancesco A. (2005). Ferritin contributes to melanoma progression by modulating cell growth and sensitivity to oxidative stress. Clin. Cancer Res..

[121] Adams P. (2008). Management of elevated serum ferritin levels. Gastroenterol. Hepatol..

[122] Hou W., Xie Y., Song X., Sun X., Lotze M.T., Zeh H.J., Kang R., Tang D. (2016). Autophagy promotes ferroptosis by degradation of ferritin. Autophagy.

[123] Mancias J.D., Wang X., Gygi S.P., Harper J.W., Kimmelman A.C. (2014). Quantitative proteomics identifies NCOA4 as the cargo receptor mediating ferritinophagy. Nature.

[124] Tang M., Chen Z., Wu D., Chen L. (2018). Ferritinophagy/ferroptosis: Iron-related newcomers in human diseases. J. Cell. Physiol..

[125] Goodall E.F., Haque M.S., Morrison K.E. (2008). Increased serum ferritin levels in amyotrophic lateral sclerosis (ALS) patients. J. Neurol..

[126] Nadjar Y., Gordon P., Corcia P., Bensimon G., Pieroni L., Meininger V., Salachas F. (2012). Elevated serum ferritin is associated with reduced survival in amyotrophic lateral sclerosis. PLoS ONE.

[127] Luo Y., Yang X., Shi Q. (2016). The cytochrome P450 inhibitor SKF-525A disrupts autophagy in primary rat hepatocytes. Chem. Biol. Interact..

[128] Zhu X., Ji M., Han Y., Guo Y., Zhu W., Gao F., Yang X., Zhang C. (2017). PGRMC1-dependent autophagy by hyperoside induces apoptosis and sensitizes ovarian cancer cells to cisplatin treatment. Int. J. Oncol..

[129] Petibone D.M., Majeed W., Casciano D.A. (2017). Autophagy function and its relationship to pathology, clinical applications, drug metabolism and toxicity. J. Appl. Toxicol..

